# Employability and career beliefs inventory: a brief version for unemployed persons

**DOI:** 10.1186/s41155-024-00309-y

**Published:** 2024-06-28

**Authors:** Ana Daniela Silva, Vinicius Coscioni, Alexandra Barros, Maria do Céu Taveira

**Affiliations:** 1 School of Psychology, University of Minho, Braga, Portugal; 2 Utrecht University, Utrecht, Netherlands; 3 Faculty of Psychology, University of Lisbon, Lisbon, Portugal

**Keywords:** Employability, Career, Validity, Reliability, Factor analysis, Measurement invariance, Graded response model

## Abstract

**Background:**

Considering that beliefs may be assessed and changed, inventories measuring employability and career beliefs may be of utmost importance for career interventions.

**Objective:**

This study introduces the psychometric properties of a brief version of the Employability and Career Beliefs Inventory (ECBI) in a sample of unemployed persons.

**Methods and results:**

Altogether, 2023 unemployed persons aged from 18 to 66 years old and living in Southern Portugal participated in an online survey. The ECBI’s original internal structure was tested and did not fit the data. Exploratory and confirmatory factor analyses were implemented, and a three-factor solution was retained. The three factors discriminate three types of beliefs named growth, pessimism, and flexibility. Measurement invariance models identified scalar equivalence across gender and educational degree, and metric invariance across age. All items fit the graded response model’s parameters. The growth and flexibility subscales were less effective in the assessment of low latent trait levels, whereas the opposite was observed with the pessimism subscale. Internal consistency is good yet discrimination between factors is questionable. Correlations to career decision-making self-efficacy evidence validity based on the relations to other constructs.

**Conclusion:**

Despite the limitations, the brief version of the ECBI proposed in this study is ready for further use and development among unemployed persons.

## Introduction

The world has been facing an economic crisis with a significant increase in unemployment rates and precarious jobs (Blustein et al., 2016). This crisis has intensified due to the Covid-19 pandemic, which has damaged economic growth (Blustein et al., 2020; Lambovska et al., 2021), especially in regions and job areas dependent on tourism (Almeida & Santos, 2020). In Portugal, where tourism plays an important economic role, the unemployment rates were above the European Union average (PORDATA, 2021). The impact was greater in the Southern touristic region named Algarve, with the rates of unemployment increased over 150% (Instituto de Emprego e Formação Profissional [IEFP], 2021). Reversing the growth of unemployment requires actions at the governmental, institutional, and individual levels. As for the individual level, interventions with unemployed persons should optimize career resources (Fugate et al., 2004; Rothwell et al., 2009). Thus, measures assessing this type of personal feature may be useful tools to assess unemployed persons. This study introduces the psychometric properties of an inventory that assesses employability and career beliefs in a sample of unemployed persons in Algarve.

### Career and employability over the past decades

Over the past decades, individual career paths have become unstable and uncertain (Akkermans et al., 2024; Krumboltz, 2009; Savickas et al., 2009; Taveira et al, 2023; Tomlinson et al., 2018). In the recent past, people often followed predictable career paths by working most of their lives in the same company. Nowadays, this type of trajectory is far from the reality of most people, with unemployment and career transitions being no longer exceptions but typical live events (Akkermans et al., 2024; Savickas et al., 2009; Savickas, 2013; Taveira et al, 2023). With the transformations in individual career paths, new theories were created to explain the changes in the labor market and people’s professional lives. The Happenstance Learning Theory (Krumboltz, 2009) states that individual career paths cannot be predicted but is rather a combination of countless planned and unplanned learning experiences. In order to achieve a satisfactory career, people must improve their cognitive, emotional, and physical skills to manage unexpected events and capitalize on opportunities. The development of these skills is related to the notion of employability.

Multiple theoretical perspectives have studied employability yet a common rationale is far from a consensus (Fuertes et al., 2021). Notwithstanding the disagreement on its definition, the current models of employability are complementary rather than contradictory (Eimer & Bohndick, 2023; Guilbert et al., 2015). Fugate et al. (2004) created a model of employability that acknowledges career identity, personal adaptability, and social and human capital as important predictors of career adaptability. The model is in line with the Life Design Paradigm, according to which career adaptability refers to individual resources to deal with career barriers and transitions (Savickas et al., 2009). After Fugate et al. (2004), countless models have expanded the notion of employability to include personal and contextual features that impact job search, maintenance, and promotion (Di Fabio, 2017; Gamboa et al, 2023; Guilbert et al., 2015; Lo Presti & Pluviano, 2015; Lo Presti et al, 2020; Van der Heijden et al., 2018).

While experiencing their professional lives, people construct employability and career beliefs that drive their behavior and how they take advantage of their skills. Thus, employability and career beliefs may either enable or debilitate people’s adaptability to career barriers and transitions (Barros, 2020). Considering that beliefs may be assessed and changed, inventories measuring employability and career beliefs may be of utmost importance for career interventions. One example is the Career Beliefs Inventory, which was created by Krumboltz (1991) grounded on cognitive therapy. It consists of a 25-subscale inventory with several subscales measured by only two items. The reduced number of items per subscale implies psychometric limitations. In addition, Barros (2020) discussed that the Career Beliefs Inventory assesses other features rather than beliefs, such as motivations, attributions, attitudes, intentions, and values. Barros (2020) created a new measure as an attempt to overcome these limitations, the Employability and Career Beliefs Inventory (ECBI).

### The Employability and Career Beliefs Inventory (ECBI)

The ECBI (Barros, 2020) is grounded on the Happenstance Learning Theory (Krumboltz, 2009), the Career Adaptivity Model of the Life Design Paradigm (Savickas et al., 2009), and the Employability Model of Fugate et al. (2004). The three models share similar views regarding human beings as agents creating their own careers and life stories. According to Happenstance Learning Theory (Krumboltz, 2009), people’s life stories are influenced by the chaos and unpredictability of the current historical time. People are seen as agents seizing opportunities by developing core skills and flexible beliefs to adjust to chaos. In Life Design Paradigm (Savickas et al., 2009), career adaptability is a key concept describing people’s ability to adapt their careers in accordance with the new demands of an unstable, unpredictable, and inconstant world. Likewise, in the Employability Model of Fugate et al. (2004), personal adaptability is a key feature to promote employability.

The test content is based on 15 interviews with managers of small to medium companies. The interviews assessed behavior and attitudes valued for the recruitment or promotion of employees. Content analyses were carried out and six dimensions were created: (1) striving/achievement, i.e., beliefs that one’s effort, persistence, and resilience are important for career goals’ attainment; (2) proactivity/initiative, i.e., beliefs that one’s own actions are important for seizing and creating career opportunities; (3) flexibility/openness to change, i.e., beliefs that one may adjust career goals to adapt to changes; (4) acceptance of challenges/risks, i.e., beliefs that one should accept career risks and challenges as opportunities to self-develop; (5) optimism, i.e., beliefs that one’s career might be successful; and (6) autonomy, i.e., beliefs that one may become more autonomous in one’s career.

The first 54-item version of the ECBI was created by three psychologists based on the six dimensions previously created. The items are expressed as statements with which participants either agree or disagree from 1 (strongly disagree) to 5 (strongly agree). After a pilot study with 38 undergraduate students, some items were rephrased. The new version was tested in a sample of 235 undergraduate students and 14 items were eliminated because they worsened the subscales’ reliability. The 38-item version was tested in a sample of 395 undergraduate students and the subscales’ reliability varied from acceptable to good, .68 ≤ *α* ≤ .86. The subscales were correlated to the five dimensions of the Dispositional Measure of Employability (Fraga, 2012; Fugate & Kinicki, 2008). The ECBI’s internal structure was tested in a principal component analysis (PCA) with Varimax rotation that suggested, according to a scree plot test, four components rather than six. The first component was comprised of items originally from the subscales flexibility and acceptance of challenges, whereas the second to fourth mostly encompassed items from the subscales striving, optimism, and proactivity, respectively. Items of autonomy are loaded both onto the first and second components.

The ECBI has some limitations. First, its theoretical internal structure was not corroborated by the PCA performed by Barros (2020). In addition, the use of PCA for assessing psychometric scales is inconsistent with psychological constructs’ measurement (Damásio, 2012). PCA reduces the items to latent variables comprised of items’ common and individual variances (i.e., the components). The aim of psychological constructs’ measures is to assess latent traits that supposedly explain beliefs, behaviors, and emotions listed in a test. Hence, the latent trait should be a latent variable that explains items’ common variance rather than items’ individual variances, i.e., a factor. Therefore, rather than PCA, factor analyses are more advised to measure psychological constructs (Costello & Osbourne, 2005). The use of varimax rotation is also controversial because orthogonal methods assume that the correlations between components are null (Damásio, 2012), which is not expected in the case of ECBI’s dimensions.

Another limitation refers to the fact that some ECBI’s items are not directly related to career nor employability. For example, Item 8 belongs to the striving/achievement dimension and is phrased like “It is important to set goals for ourselves and make an effort to achieve them.” Even though the item expresses striving and achievement, it does not address career issues. The ECBI is a thematic measure focused on a specific life domain. Thus, the inclusion of items unrelated to career may raise doubts regarding its content validity.

Not as much as a limitation but still noteworthy, the ECBI psychometric properties were extracted from studies carried out with convenient samples of undergraduate students. Therefore, they cannot be generalized to community samples and nor specific samples rather than undergraduate students. Lastly, the ECBI contains many items, which may considerably enlarge the time of data collection. Short versions with less redundant content may be proposed to optimize the time and quality of data collection. Even though the ECBI has several limitations, its content assesses important facets of career and employability beliefs. Therefore, the literature may be benefited from studies improving the ECBI as well as testing it in other samples rather than undergraduate students.

This article introduces the psychometric properties of a brief version of the ECBI in a sample of unemployed persons. The specific goals were as follows: (1) to extract the ECBI’s internal structure based on exploratory and confirmatory factor analyses; (2) to test the invariance of the ECBI’s factor structure, factor loadings, and intercepts across gender, age, and educational degree; (3) to identify the discrimination and difficulty of the ECBI’s items based on Graded Response Model; (4) to assess the adequacy of the ECBI’s rating scale; (5) to assess the range of latent trait level best assessed by the ECBI; (6) to identify the ECBI’s factors’ reliability; and (7) to identify validity evidence based on the association with a related measure.

For the assessment of validity evidence based on the association with related measures, the Career Decision-Making Self-Efficacy Scale was elected. The choice stems from previous studies suggesting that the strengthening of enabling career and employability beliefs may have beneficial effects on self-efficacy beliefs (Berntson et al., 2008) as well as that self-efficacy beliefs may predict employability resources (Ahmed et al., 2019; Taveira et al., 2017) and other career prospects (Chow et al., 2019; Wujema et al., 2022). Considering that employability and career beliefs may either enable or debilitate career resources (Barros, 2020), it is expected that career decision-making self-efficacy is positively associated with enabling employability and career beliefs.

## Method

### Participants and procedures

All persons registered as unemployed in governmental institutions in Algarve with an e-mail contact (*N* = 10,272) were invited to participate in an online survey in SurveyMonkey. Altogether, 2066 persons responded to the questionnaires, of which 43 were eliminated for they filled in the same response category for all items. Hence, 2023 answers were analyzed. Participants were aged from 18 to 66 years old (*M* = 39.7, *SD* = 10.87) and were predominantly female (*n* = 1348, 66.6%). Regarding their educational degree, 585 (28.9%) had not finished high school, 846 (41.8%) had completed high school, and 592 (29.3%) had a college degree.

Data collection occurred for six days in April 2021, at the beginning of an isolation period in Portugal due to the Covid-19 pandemic. The data were collected within the framework of the Careers Project—workshops for employability (ALG-06-4234-FSE-000047), a partnership for social impact that aims to develop an intervention to support employability in Algarve. Participants provided their consent before answering the questionnaires, and the research study was approved by an ethical commission from Portugal (*CEICSH 002/2022*).

### Additional measure

In addition to the ECBI, participants filled in the Career Decision-Making Self-Efficacy Scale, originally created in the USA (Lent et al., 2016) and already adapted to Portugal (Lent et al., 2021). The scale contains eight items answered on a 5-point scale varying from 1 (totally disagree) to 5 (totally agree). Its internal structure fit well the data, *χ*^2^ = 1387.055, *df* = 20, *p* < 0.001, *RMSEA* = .184 [10% C.I. = .176; .192], *CFI* = .968, *TLI* = .955, *SRMR* = .050,^1^ and the reliability was excellent, *α =* .95 and *Ω =* .92.

### Data analysis

#### Data exploration

Before assessing the ECBI’s psychometric properties, data were explored. Multivariate outliers were identified by Mahalanobis distance, with the cutoff being *M/df* < 4.0 (Hair et al., 2009). Univariate and multivariate normality were tested via Shapiro-Wilk and Mardia’s tests.

#### Factor analyses

The ECBI’s internal structure was assessed via exploratory factor analyses (EFA) and confirmatory factor analyses (CFA). Weighted least squares mean and variance adjusted (WLSMV) estimation method (Asparouhov & Muthen, 2010) and polychoric correlation matrix were used because variables were considered ordinal and violated normality. Before the EFA, the adequacy of the correlation matrix was examined via the Kaiser-Meyer-Olkin test and Bartlett’s test. Two methods were compared to decide on the number of factors to retain, i.e., Kaiser criterion (i.e., Eigenvalues ≥ 1.0) and Parallel Analyses (Timmerman, & Lorenzo-Seva, 2011). Closeness to unidimensionality was assessed by three indices—i.e., Unidimensional Congruence (UniCo), Explained Common Variance (ECV), and Mean of Item Residual Absolute Loadings (MIREAL), with the cutoffs being *UniCo* ≤ .95, *ECV* ≤ .85, and *MIREAL* ≥ .30 (Ferrando & Lorenzo-Seva, 2018). The retained factors were rotated with Direct Oblimin. For the CFA, modification indices were assessed to refine the internal structure.

The following fit indices were computed to assess the goodness of fit of the EFA and the CFA: Comparative Fit Index (CFI), Tucker-Lewis Index (TLI); root mean square error approximation (RMSEA), and standardized root mean residual (SRMR). The following cutoffs were used to indicate good fit: *CFI* ≥ .95, *TLI* ≥ .95, *RMSEA* < .08, and *SRMR* ≤ .08 (Schreiber et al., 2006). Alternatively, the following cutoffs were considered acceptable: .90 ≤ *CFI* < .95, .90 ≤ *TFI* < .95, .080 ≥ *RMSEA* > .100, and .080 > *SRMR* ≥ .100 (Brown, 2006).

#### Measurement invariance testing

Multigroup CFA were conducted to assess measurement invariance across gender, age, and educational degree. As some of the response categories were not filled in by participants from all groups, WLSMV estimation method was not allowed. Therefore, the Pearson correlation matrix and maximum likelihood robust (Satorra & Bentler, 2001) estimation method were used. transgender and gender non-conforming participants were not considered in the assessment of invariance across gender due to the reduced number of participants belonging to these categories (*n* = 5). For the assessment of invariance across age, three age groups were considered: 18 to 30 years old, 31 to 45 years old, and 46 to 66 years old. Configural, metric, and scalar models were computed, which respectively assessed whether the proposed factor structure, factor loadings, and intercepts were equivalent across the groups. Invariance was assumed only if no big differences were observed across models (configural versus metric, and metric versus scalar), i.e., *ΔRMSEA* ≤ .015, ΔSRMR ≥ .015 (or .030 in metric invariance) and *ΔCFI* ≥ −.01 (Chen, 2007; Cheung & Rensvold, 2002).

#### Graded response model

Items were tested through graded response model (Samejima, 1969), which is an Item Response Theory model for polytomous data. Each factor was tested separately. The frequency of answers in each response category per item was assessed because low rates of responses may decrease the accuracy of item parameters’ estimation (Toland, 2014). Thus, categories with 20 or fewer responses (1% of participants) were merged.

Before interpreting GRM parameters, statistical assumptions and fit indices were tested. Previous results of CFA and EFA were considered evidence of unidimensionality. Local dependence was assessed by the *Q*_*3*_ test and values below |1/(*L*–1)| were expected—with *L* meaning the length of the scale (Yen, 1993). Absolute values below .224 were also accepted no matter the length of the scale (de Ayala, 2009). Monotonicity was assessed by scalability coefficient *H*, with values above .30 being expected (Mokken, 1971). As for the fit indices, the overall model was tested by *C*_*2*_ test. Even though *M*_2_* test is more appropriate for polytomous data, it is not allowed for models with reduced degrees of freedom (Paek and Cole, 2019). Item and person fit were tested by *S-χ*^*2*^ test and *Zh* statistic, respectively. *C*_*2*_ and *S-χ*^*2*^ tests assess the degree of similarity between predicted and empirical models, with non-significant values suggesting that items’ observed response frequencies fit the GRM parameters (Toland, 2014). Conversely, Cook et al. (2009) discussed that *p-*values of *C*_*2*_ and *S-χ*^*2*^ tests are impacted by several features such as the length of the scale and the sample size. Based on that, CFA fit indices were used to inform the model and item fit (Cook et al., 2009). As for the *Zh* statistic, values below −3.0 suggest potential aberrant response patterns (Paek & Cole, 2019). Finally, the reliability of participants’ latent trait levels was assessed by the *Rho* coefficient, with values above 0.70 being expected (Sijtsma & Molenaar, 1987).

After testing assumptions and fit indices, GRM logistic parameters were interpreted. Item discrimination (*a*) informs the degree to which the responses in an item can distinguish participants with different latent trait levels (*ɵ*). The following cutoffs were used to interpret the level of discrimination: values below .64 are low; between .65 and 1.34 are moderate; between 1.35 and 1.69 are high; and above that, very high (Baker & Kim, 2017). GRM estimates *K*-1 item difficulty thresholds, being *K* the number of response categories. Item difficulty (*b*) suggests the *ɵ* at which one has the same chance to endorse two distinct response categories. For instance, *b*_*1*_ indicates the *ɵ* at which one has the same chance to fill in the first or second response categories; *b*_*2*_ indicates the *ɵ* at which one has the same chance to fill in the second or third response categories; and so on. General item difficulty parameters (*b*) were also extracted with high values suggesting that the item requires more *ɵ* to endorse higher response categories. In addition to assessing item parameters, item characteristic curves (ICC) and test information curves (TIC) were plotted to appraise the adequacy of the rating scale and the range of *ɵ* best assessed by the test.

#### Reliability

Three internal consistency coefficients tested subscales’ reliability, i.e., Cronbach’s ordinal alpha (α), McDonald’s omega (Ω), and Spearman-Brown coefficient (*r*_*kk*_). The following cutoffs were used to inform the level of reliability: values below .50 are unacceptable; between .51 and .60 are poor; between .61 and .70 are questionable; between .71 and .80 are moderate; between .81 and .90 are good; and above that, excellent (Gliem & Gliem, 2003). In addition, average variance extracted (AVE) was assessed and values above .50 were expected (Fornell & Larcker, 1981).

#### Correlations between factors and to career decision-making self-efficacy

Finally, the associations between the ECBI’s factors and the Career Decision-Making Self-Efficacy Scale were assessed via Spearman correlations. The correlations were computed considering the factor scores extracted with the maximum a posteriori method (Bock & Aitkin, 1981). The following cutoff values were used to indicate the magnitude of correlations: values below .30 are weak; between .31 and .70 are moderate; and above that, strong (Dancey & Reidy, 2007). In addition, we expected correlation coefficients below the root square of factors’ AVE, which evidences that factors are distinguishable (Fornell & Larcker, 1981).

#### Adequacy of sample size

The sample size was adequate for all analyses. Considering the EFA, the sample size was above 250 (Catell, 1978), and the ratio of participants per item was greater than 10 (Everitt, 1975). For the CFA, the sample size was above 200 as well as bigger than five times the number of parameters in the tested models (Kline, 2011). Lastly, for the GRM, the sample size was greater than 500 (Nunes & Primi, 2005).

#### Software programs

All analyses were carried out in R 4.1.1 (R Core Team, 2020), except for the EFA, which were carried out in the software FACTOR (Lorenzo-Seva & Ferrando, 2006). Several packages were used in R: multivariate normality was tested by MNV 5.9 (Korkmaz et al., 2014); CFA were performed with lavaan 0.6-9 (Rosseel, 2012); reliability was assessed by semTools 0.5-5 (Jorgensen et al., 2021), psych 2.3.3 (Revelle & Revelle, 2015), and multicon 1.6 packages (Sherman, 2015); GRM was implemented with mirt 1.34 (Chalmers, 2012); and scalability coefficient H was tested by Mokken 3.0.6 (van der Ark et al., 2021).

## Results

### Data exploration

Data exploration indicated adequacy of the number of multivariate outliers and violation of normality. Only 18 participants (0.9%) were outliers (i.e., *M/df* > 4.0), and no reasons for their elimination were acknowledged. Mardia’s test indicated violation of multivariate normality, *M*_*skewness*_* =* 43,203.914, *p* < .001, and *M*_*kurtosis*_ = 227.857, *p* < .001. In turn, Shapiro-Wilk tests considering each item separately were all significant at .001, which indicates a violation of univariate normality.

### Factor analyses

#### Original factor structure testing

The ECBI’s original internal structure was assessed by a CFA that resulted in poor fit, *χ*^2^ = 8786.829, *df* = 650, *p* < 0.001, *RMSEA* = .079 [10% C.I. = .077; .080], *CFI* = .876, *TLI* = .865, *SRMR* = .064. Next, the 4-factor solution considering the findings of the PCA performed by Barros (2020) was tested, with results also indicating poor fit, *χ*^2^ = 9421.097, *df* = 659, *p* < 0.001, *RMSEA* = .081 [10% C.I. = .080; .083], *CFI* = .866, *TLI* = .857, *SRMR* = .067. As neither the theoretical nor the empirical model proposed by Barros (2020) fit the data, we moved to exploratory mode as an attempt to extract a new factor structure able to fit the data and properly explain career and employability beliefs. Thus, the sample was randomly divided into two halves; EFA were conducted with the first (*n* = 1012) and CFA with the second (*n* = 1011).

#### Exploratory factor analyses

The sample was randomly divided into two halves, and EFA were conducted with the first one (*n* = 1012). Kaiser-Meyer-Olkin test and Bartlett’s test were conducted and indicated very good adequacy of the correlation matrix, *KMO* = .95, and *χ*^2^ = 11,484.1, *df* = 703, *p* < .001, respectively. Kaiser criterion and Parallel Analysis suggested the retention of six and two factors, respectively. Two closeness to unidimensionality indices suggested that the scale is not unidimensional, *UniCo* = .885 and *ECV* = .840, whereas one suggested that it is, *MIREAL* = .197. The 6-factor solution resulted in several items with double factor loadings greater than .30 as well as several items with no factor loadings over .30. The same pattern was observed for the 5-factor and 4-factor solutions. The 3-factor solution formed one factor hardly interpretable that contained most of the items. Lastly, the 2-factor solution resulted in one factor with all direct items and another with all reverse-coded items.

The test content was assessed by two experts in the field working together aiming to remove items not directly related to career or employability. As discussed in the introduction, some ECBI’s items refer to general motivational features rather than career or employability, e.g., “It is important to set goals for ourselves and make an effort to achieve them.” The two experts worked together to assess the items and decided on the elimination of 15 items that were not directly related to career or employability^2^. New analyses were carried out with the 23 remaining items. Kaiser criterion and Parallel Analysis suggested the retention of three and two factors, respectively. UniCo and ECV indicated that the scale is not unidimensional, *UniCo* = .872 and *ECV* = .823, whereas MIREAL suggested that it is, *MIREAL* = .215. The 2-factor solution resulted, again, in one factor with all direct items and another with all reverse-coded items. The 3-factor solution was the most theoretically interpretable and thus chosen to be refined.

Five items were eliminated for they had two or more factor loadings with differences lower than .20. With the elimination of the five items, Kaiser criterion, Parallel Analysis, and the closeness to unidimensionality indices, *UniCo* = .838, *ECV* = .798, and *MIREAL* = .237, kept the same suggestions as in the 3-factor solution with the 23 items. The 3-factor solution with 18 items presented excellent fit indices, *RMSEA* = .026, *CFI* = .992, *TLI* = .994. Table 1 depicts the factor loadings, eigenvalues, proportion of common variance, and reliability coefficients. All factor loadings were greater than .50, the three factors altogether explained 58.5% of the variance, and the reliability coefficients varied from moderate to excellent.

**Table 1:** Exploratory factor analysis

	Translated items	Factor 1 (growth)	Factor 2 (pessimism)	Factor 3 (flexibility)
4.	There are so few vacancies per applicants that getting an opportunity is almost impossible	.106	**.611**	−.056
5.	Sometimes, accepting a job that is not the ideal one may lead to promising new opportunities	−.129	−.073	**.684**
11.	I am seeking to evolve professionally to work with great autonomy and responsibility	**.752**	−.037	.005
13.	If a professional experience goes wrong, we should learn from our mistakes and do it better	**.709**	.024	.100
15.	Accepting a challenging job may be an opportunity to develop skills and abilities	**.584**	.019	.215
18.	Even if a job is temporary or uncertain, it is worth taking it because it is an opportunity to have new experiences	.103	−.007	**.639**
19.	One day I will be able to do my work with plenty of autonomy	**.784**	−.068	-.056
20.	My professional future might be rough	.058	**.689**	-.084
21.	It is important to develop our skills to the fullest and always improve as a professional	**.772**	−.079	-.020
22.	It is not worthy sending curricula to companies because one never gets an answer	−.051	**.542**	-.064
24.	Even if a professional situation is not ideal, it is always an opportunity to learn something	.241	−.028	**.622**
26.	It is better not to have high professional expectations so that we are not disappointed for not meeting them	−.077	**.620**	.109
27.	In order to progress in career, one must take gradually bigger responsibilities	**.654**	.058	.040
28.	Professional challenges and barriers help us improving ourselves	**.633**	.005	.202
31.	A good way to build our future is to take initiative in finding or creating our own job opportunities	**.521**	−.105	.102
32.	Even if a job does not match what we want, accepting it may be an opportunity to prove what we are capable of	.025	.022	**.802**
34.	When I do not succeed in school or work, I think I am not capable	−.196	**.575**	.076
38.	One day I want to be able to do my work with autonomy	**.785**	.052	−.109
		Factor 1 (growth)	Factor 2 (pessimism)	Factor 3 (flexibility)
Eigenvalue		6.93	2.12	1.49
% variance		38.5%	11.8%	8.3%
*α*		.91	.76	.81
Spearman correlations		Factor 1 (growth)	Factor 2 (pessimism)	Factor 3 (flexibility)
Factor 1 (growth)		1.00	−.394*	.549*
Factor 2 (pessimism)			1.00	−.194*
Factor 3 (flexibility)				1.00

*n* = 1012, *p < .05

The three factors integrated nine, five, and four items and were named growth, pessimism, and flexibility. Growth is composed of items originally from autonomy (four items), striving/achievement (two items), acceptance of challenges/risks (two items), and proactivity/initiative (one item). It is related to positive beliefs that one may flourish while dealing with career barriers. Pessimism is encompassed by four reverse-coded items originally from optimism and one reverse-coded item originally from proactivity/initiative. It entails negative beliefs that one might not succeed in one’s career. Lastly, flexibility is formed by three items originally from flexibility/openness to change and one item originally from acceptance of challenges/risks. It pertains to beliefs that one may follow unexpected career paths as a means to take advantage of opportunities. Growth was positively correlated to flexibility and negatively correlated to pessimism, with the magnitude of the correlations being moderate. Flexibility was correlated negatively and weakly to pessimism (Table 1).

#### Confirmatory factor analyses

**Fig. 1:**
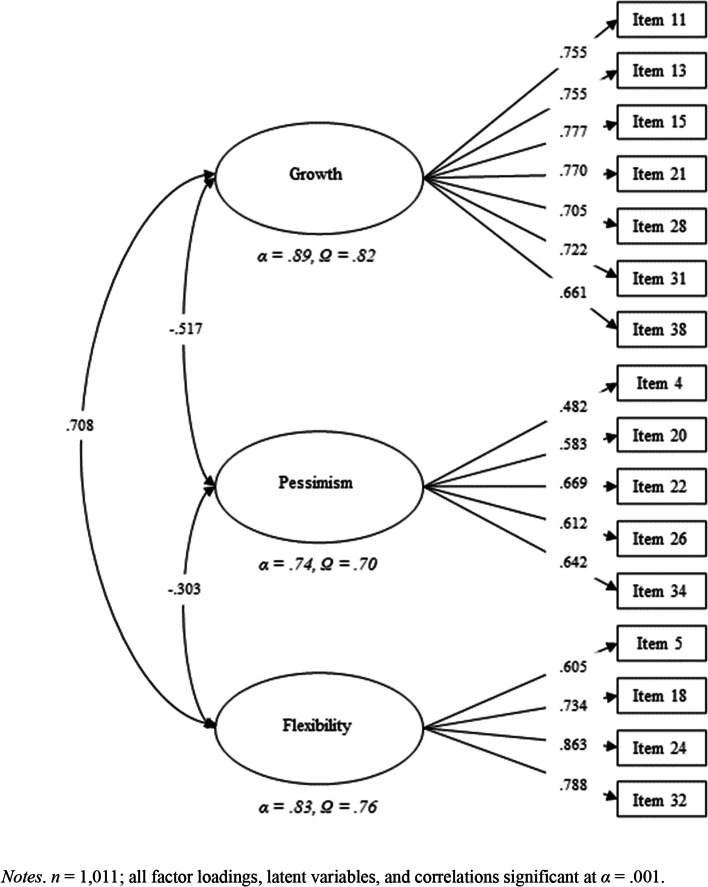
Confirmatory factor analysis. Note: *n* = 1011; all factor loadings, latent variables, and correlations are significant at *α* = .001

The internal structure proposed after the EFA was tested in a CFA with the second half of the sample. The internal structure fit the data, *χ*^2^ = 793.167, *df* = 132, *p* < 0.001, *RMSEA* = .070 [10% C.I. = .066; .075], *CFI* = .954, *TLI* = .947, *SRMR* = .051. Conversely, modification indices suggested high correlations between three item pairs. Two pairs belonged to the growth subscale—i.e., items 19 (“One day I will be able to do my job with complete autonomy”) and 38 (“One day, I want to be able to develop my work independently”); and items 27 (“To evolve professionally, it is important to be able to take on increasingly greater responsibilities.”) and 28 (“Professional challenges and difficulties help us to be better”). The third pair belonged to the pessimism subscale—i.e., items 4 (“There are so few jobs for so many candidates that it is almost impossible to get an opportunity”) and 20 (“My professional future will be very difficult”). The high correlation between the two item pairs in growth subscale might be associated with content redundancy. Therefore, a new model was fitted excluding items 19 and 27, which had the lowest factor loadings in their pairs. No items of the pessimism subscale were eliminated because that would have worsened the reliability. Moreover, the content of these two items was not redundant. After the elimination of the two items, fit indices improved, *χ*^2^ = 469.226, *df* = 101, *p* < 0.001, *RMSEA* = .060 [10% C.I. = .055; .066], *CFI* = .969, *TLI* = .963, *SRMR* = .045. Figure 1 exhibits the final factor structure, with its factor loadings, reliability, and correlations between factors. All factor loadings were greater than .50, except for item 4. Reliability ranged from questionable to good, and correlations between factors varied from moderate to strong.

### Measurement invariance models

Table 2 depicts the fit indices of the configural, metric, and scalar invariance models across gender, age, and educational degree considering the entire sample. The findings indicated scalar invariance across gender and educational degree, and metric invariance across age. Partial invariance models identified scalar variance only across the first and third age groups, particularly regarding the intercepts of items 22 and 34, both from the pessimism subscale. Item 22 intercept was greater in the third age group, whereas item 34 intercept was greater in the first.

**Table 2:** Invariance measurement models

	*χ*^*2*^(*df*)	*RMSEA* [CI 90%]	*CFI*	*TLI*	*SRMR*	*Δχ*^*2*^(*Δdf*)	*ΔCFI*	*ΔRMSEA*	*ΔSRMR*
Gender (females, *n* = 1348; males, *n* = 673)									
Configural	442.5 (202)*	.034 [.030; .038]	.961	.953	.037				
Metric	453.0 (215)*	.033 [.029; .037]	.961	.957	.038	10.5 (13)	.000	−.001	.001
Scalar	515.4 (228)*	.035 [.032; .039]	.953	.951	.040	62.4 (13)*	.008	.002	.002
Qualification (elementary school, *n* = 585; high school, *n* = 846; college degree, *n* = 592)									
Configural	578.8 (303)*	.037 [.033; .041]	.956	.948	.040				
Metric	608.0 (329)*	.035 [.032; .039]	.955	.951	.044	29.2 (26)*	−.001	−.002	.004
Scalar	664.2 (355)*	.036 [.032; .040]	.951	.950	.045	56.2 (26)*	−.004	.001	.001
Age (18–30 years, *n* = 490, 31–45 years, *n* = 908, 46–66 years, *n* = 620)									
Configural	592.4 (303)*	.038 [.034; .042]	.954	.946	.041				
Metric	629.7 (329)*	.037 [.033; .041]	.953	.948	.045	37.3 (26)*	−.001	−.001	.004
Scalar	761.2 (355)*	.041 [.038; .045]	.936	.941	.049	131.5 (26)*	−.017	.004	.004
Age—groups 1 and 2 (18–30 years, *n* = 490, 31–45 years, *n* = 908)									
Configural	413.2 (202)*	.039 [.034; .044]	.954	.945	.041				
Metric	423.8 (215)*	.037 [.032; .042]	.955	.949	.042	10.6 (13)	.001	−.002	.001
Scalar	481.5 (228)*	.040 [.035; .044]	.945	.942	.044	57.7 (13)*	−.010	.003	.002
Age–groups 2 and 3 (31–45 years, *n* = 908, 46–66 years, *n* = 620)									
Configural	418.8 (202)*	.037 [.033; .042]	.955	.946	.040				
Metric	439.4 (215)*	.037 [.033; .041]	.951	.945	.044	20.6 (13)	−.004	.000	.004
Scalar	483.1 (228)*	.038 [.034; .042]	.947	.944	.045	143.7 (13)*	−.004	.001	.001
Age—groups 1 and 3 (18–30 years, *n* = 490, 46–66 years, *n* = 620)									
Configural	352.5 (202)*	.037 [.031; .042]	.955	.946	.043				
Metric	379.6 (215)*	.037 [.032; .043]	.951	.945	.048	27.1 (13)*	−.004	.000	.005
Scalar	487.5 (228)*	.045 [.040; .050]	.922	.918	.054	107.9 (13)*	−.029	.008	.006
Partial^1,2^	420.1 (226)*	.039 [.034; .045]	.942	.938	.050	40.5 (11)*	−.009	.002	.002

^*^p < .05, ^1^intercepts of items 22 and 34 not fixed, ^2^Δχ^2^(Δdf) and ΔCFI were computed considering the metric model

### Graded response model

For the estimation of GRM parameters, some response categories were merged. In the growth subscale, a reduced number of participants (*n* < 20) filled in the two first response categories and, therefore, the three first answer options were merged. Hence, only two thresholds (*b*_*3*_ and *b*_*4*_) were estimated for the growth subscale, whereas the pessimism and flexibility subscales had four thresholds estimated (*b*_*1*_ to *b*_*4*_).

GRM assumptions were partially observed. CFA and EFA evidenced the subscales’ unidimensionality. Scalability coefficients *H* were all above .30 (Appendix 1), which suggests preservation of monotonicity. As for local dependence, the *Q*_*3*_ test revealed that all residual correlations in the growth subscale were below .224 despite eight values above 1/(*L*-1), i.e., |.166|. In the pessimism subscale, three item pairs had residual correlations above .224, of which only two exceeded 1/(*L*-1), i.e., |.250|. In the flexibility subscale, two item pairs had residual correlations above .224 yet only one exceeded 1/(*L*-1), i.e., |.333|. Thus, three item pairs violated both criteria for local dependence, which suggests biases in the estimation of item parameters in pessimism (two pairs, 20%) and flexibility (one pair, 16.7%) subscales.

Appendix 1 exhibits RMSEA results of *S-χ*^*2*^ test, and Appendix 2 depicts the fit indices of *C*_*2*_ test as well as information regarding person fit. *S-χ*^*2*^ tests achieved all excellent RMSEA values except for item 28, which achieved only acceptable results. *C*_*2*_ tests met excellent fit indices, except for RMSEA of the pessimism subscale, which is slightly above .060. *Zh* statistics below −3.0 were observed for less than 5% of respondents across the three subscales, which indicates a good person fit. Lastly, all *ρ* reliability coefficients were above .70, as expected.

Table 3 exhibits the GRM item parameters. In the growth subscale, all items had very high discrimination but item 31, which achieved only a high cutoff. Item 21 was the most discriminant one, whereas items 13 and 31 were the least and the most difficult ones, respectively. In the pessimism subscale, items 20, 26, and 34 had high discrimination, with item 20 being the most discriminant one. Items 4 and 22 had moderate discrimination and were the least and the most difficult ones, respectively. In the flexibility subscale, all items had very high discrimination except for item 5, which achieved only a high cutoff. Item 32 was the most discriminant and difficult one, whereas item 24 was the least difficult one.

**Table 3:** GRM—logistic parameters

	Translated items	Logistic parameters					
		*a*	*B*	*b*_*1*_	*b2*	*b3*	*b4*
Growth							
11	I am seeking to evolve professionally to work with great autonomy and responsibility	2.36	−1.03	---	---	−2.22	0.17
13	If a professional experience goes wrong, we should learn from our mistakes and do it better	2.26	−1.24	---	---	−2.49	0.01
15	Accepting a challenging job can be an opportunity to develop skills and competences	1.92	−0.81	---	---	−2.10	0.48
21	It is important to develop our skills to the fullest and always improve as a professional	2.64	−1.06	---	---	−2.20	0.07
28	Professional challenges and barriers help us improving ourselves	1.91	−0.60	---	---	−1.83	0.63
31	A good way to build our future is to take initiative in finding or creating our own job opportunities	1.70	−0.41	---	---	−1.77	0.95
38	One day I want to be able to do my work with autonomy	1.72	−0.99	---	---	−2.28	0.29
Pessimism							
4	There are so few vacancies per applicants that getting an opportunity is almost impossible	1.32	0.03	−2.11	−0.53	0.57	2.25
20	My professional future might be rough	1.70	0.37	−1.55	−0.30	1.00	2.48
22	It is not worthy sending curricula to companies because one never gets an answer	1.32	1.18	−1.18	0.66	1.82	3.09
26	It is better not to have high professional expectations so that we are not disappointed for not meeting them	1.36	0.50	−1.66	0.00	0.96	2.89
34	When I do not succeed in school or work, I think I am not capable	1.45	1.46	−0.91	0.94	2.00	3.75
Flexibility							
5	Sometimes, accepting a job that is not the ideal one may lead to promising new opportunities	1.42	−1.57	−3.70	−2.47	−0.81	1.65
18	Even if a job is temporary or uncertain, it is worth taking it because it is an opportunity to have new experiences	1.96	−1.65	−3.21	−2.29	−1.13	0.85
24	Even if a professional situation is not ideal, it is always an opportunity to learn something	2.57	−1.93	−3.39	−2.54	−1.38	1.02
32	Even if a job does not match what we want, accepting it may be an opportunity to prove what we are capable of	2.80	−1.39	−2.71	−1.94	−0.90	1.09

*n* = 2023

Appendix 3 portrays the TICs of the three subscales. Growth and flexibility best assess latent trait levels from −4.0 to 2.0, both with a decline of the curve in the medium region of the graph. Conversely, the pessimism subscale has a more continuous curve indicating the best assessment of latent trait levels from −2.0 to 4.0. Therefore, the growth and flexibility subscales are less effective in the discrimination of participants with high levels of the latent traits, whereas the opposite occurs with the pessimism subscale. Lastly, each ICC was separately assessed (Appendix 4). The 5-point scale seemed adequate for the pessimism and flexibility subscales, whereas the 3-point scale seemed adequate for the growth subscale, with the three first response categories merged.

### Descriptive statistics and reliability

Table 4 depicts the descriptive statistics of factors’ direct scores and internal consistency coefficients. The mean of the growth subscale’s direct scores suggests a ceiling effect and thus corroborates the results of GRM. The three internal consistency coefficients (*α*, *Ω*, and *r*_*kk*_) indicated moderate to good reliability. AVE was adequate for the growth and flexibility subscales, whereas the pessimism subscale’s AVE was below the expected value of .5.

**Table 4:** Descriptive statistics, reliability, and correlations between factors and to career decision-making self-efficacy

Subscale	Direct scores	Reliability					Correlations			
	*M* (*SD*)	*α*	Ω	*r*_kk_	AVE	$\sqrt{\text{AVE}}$	Grow	Pess	Flex	CDMSE
Growth	4.3 (0.42)	.89	.82	.81	.53	.73	1.0*	−.59*	.78*	.51*
Pessimism	2.6 (0.72)	.75	.71	.70	.37	.61	−.59*	1.0*	−.38*	−.41*
Flexibility	3.9 (0.57)	.82	.75	.76	.54	.73	.78*	−.37*	1.0*	.37*

*n* = 2023, *p < .05, *CDMSE* career decision-making self-efficacy

### Correlations between factors and to career decision-making self-efficacy

Table 4 also exhibits correlations between factors and to career decision-making self-efficacy. Correlations between the growth and flexibility subscales were above the root square of their AVE, which may suggest that these factors are indistinguishable. Career decision-making self-efficacy was positively correlated to growth and flexibility as well as negatively correlated to pessimism, with the magnitude of correlations being moderate.

## Discussion

This article introduces the psychometric properties of a brief version of the ECBI, particularly in a sample of unemployed persons. Data collection was online and included a sample of 2023 unemployed persons in Algarve, Southern Portugal. A CFA indicated that the ECBI’s original internal structure did not fit the data, and therefore, a new internal structure was proposed grounded on EFA and CFA. Rather than 38 items and six factors, the proposed internal structure is comprised of 16 items and three factors. Growth (seven items) refers to positive beliefs that one may flourish while dealing with career barriers. Pessimism (five items) entails negative beliefs that one might not succeed in one’s career. Lastly, flexibility (four items) pertains to beliefs that one may follow unexpected career paths as a means to achieve new opportunities.

The invariance of the new version was tested considering different groups of gender, age, and educational degree. The findings suggested scalar invariance across gender and educational degree. Conversely, the findings suggested invariance only at the metric level across age, with differences in the intercepts of two items of the pessimism subscale (items 22 and 34) across participants aged between 18 and 30 years, and 45 and 66 years. According to Vandenberg and Lance (2000), “intercept differences may not reflect biases (undesirable) but response threshold differences that might be predicted based on known group differences (desirable)” (p. 38). The greater intercept of item 22 in the third group indicates that older participants were more likely to answer the item in accordance with other features rather than pessimistic beliefs. Item 22 refers to the expectancy of obtaining responses from companies after sending curricula. The intercept difference might be related to the fact that older unemployed persons are more likely to face barriers while obtaining responses from companies during job searches (Axelrad et al., 2018). Likewise, the greater intercept of item 34 in the first age group suggests that younger participants were more likely to respond to the item with the influence of other features rather than pessimistic beliefs. Item 34 entails the impact of school and work failure on self-efficacy. The literature suggests that self-efficacy increases with age, which often impacts how one deals with one’s professional life (Wickstrøm et al., 2020). Thus, age differences on self-efficacy might explain intercept differences in item 34. Even though the intercept differences may be explained in terms of desirable known group differences, they can still limit comparisons across younger and older participants. In such situations, differences across group may be related to differences at the level of intercepts rather than the scores.

The findings suggested that the three subscales fit the parameters of GRM. All 16 items are good discriminant of what they are supposed to assess, especially those of the growth and flexibility subscales. Items in the pessimism subscale were more difficult than those in the flexibility and growth subscales. The test information curves corroborated the results, with the growth and flexibility subscales being less effective in the assessment of high latent trait levels, whereas the pessimism subscale was less effective in the assessment of low latent trait levels. The different patterns across scales are related to the valence of their items. While growth and flexibility capture positive career beliefs, pessimism reflects negative ones. The item characteristic curves indicated that the 3-point rating scale (merging the three first response categories) fit the growth subscale, whereas the 5-point rating scale fit both the pessimism and flexibility subscales. Merging the three first response categories of the growth subscale stems from the low frequency of answers in the two first response categories, which, aligned with the high mean of direct scores, suggests a ceiling effect. This consists of a limitation of the current form, which is apparently less effective in the distinction between participants with medium to high rates of growth beliefs. Future studies could investigate whether the 3-point scale would work better. Furthermore, new versions of the ECBI should consider the inclusion of items able to distinguish medium to high latent trait levels of growth and flexibility, as well as low to medium latent trait levels of pessimism.

The subscales’ reliability was assessed through three coefficients (*α*, *Ω*, and *r*_*kk*_) that indicated moderate to excellent internal consistency. Conversely, the pessimism subscale’s AVE was below 0.5, which may suggest questionable discriminant validity (Fornell & Larcker, 1981). In addition, the comparison of AVE and correlation coefficients suggested that the growth and flexibility subscales are indistinguishable. The correlation between the factors exceeded only slightly the square root of AVE. Therefore, there is little evidence against the outperformance of the 3-factor solution in comparison to the 2-factor one. The findings mostly suggest that comparisons across growth and flexibility beliefs may be impacted by the high correlations between the two factors. Future versions of the ECBI should be composed of new items created since the first steps in accordance with the new proposed internal structure, which might optimize discrimination across factors.

The correlations between the ECBI’s factor scores to career decision-making self-efficacy consist of validity evidence based on the associations with related measures. Growth and flexibility were positively associated with career decision-making self-efficacy, whereas negative correlations to pessimism were identified. The findings are in line with the literature suggesting that enabling employability and career beliefs associate with career resources (Barros, 2020), whereas the opposite is consequently expected of debilitating beliefs.

The 3-factor solution proposed in this study substantially differs from the 6-dimension theoretical structure and the 4-dimension empirical structure described by Barros (2020). The differences might be related to the statistical methods applied across studies. Barros (2020) examined the ECBI’s internal structure by PCA with varimax rotation, which is inconsistent with the measurement of psychological constructs (Damásio, 2012). Conversely, in this study, we used more proper methods, and thus, the results are more valid and reliable.

The differences might also associate with the exclusion of 15 items not directly related to career nor employability. The exclusion of these items aimed at more content validity because the EBCI is a thematic measure focused on a specific life domain rather than a general athematic scale. The 15 items were unequally excluded across the six theoretical dimensions, with dimensions losing one to five items. Different results could have been found if the six theoretical dimensions had a similar number of items. Nevertheless, the EFA with all items already resulted in three factors with an Eigenvalue above 1.0.

The nature of the data collection might also explain the differences across the internal structures. Barros (2020) carried out the data collection with undergraduate students before the Covid-19 pandemic, whereas this study’s data collection occurred with unemployed persons in Algarve during the pandemic. Future studies still need to test the measurement invariance of the internal structure proposed in this study across different types of samples, such as undergraduate students and employees.

Notwithstanding the proposed internal structure is not equivalent to the theoretical one initially hypothesized by Barros (2020); its three factors are theoretically interpretable and insightful for future practices. The current version of the ECBI is a short and parsimonious instrument useful for research and practice with unemployed people. In the research context, it might be used in investigations interested in employability and career beliefs. In the professional context, the EBCI might be applied in counseling practices to help identifying beliefs impacting how unemployment is lived. Based on its scores, interventions may be designed to directly support career decision-making processes.

## Limitations

This study has limitations regarding the data collection and participants’ recruitment. First, it consists of an online data collection, and thus, future studies may be carried out in more controlled environments. Second, a large extent of participants had not finished high school, which might have impacted the questionnaires’ comprehension. Third, some participants were foreigners, though no question regarding their nationality was included. Participants’ nationality might have impacted the results due to language comprehension and cultural issues. Fourth, only unemployed persons with an e-mail contact were recruited, which is an undesired recruitment bias because participants without an e-mail contact did not have the chance to participate. Lastly, participants were recruited by governmental institutions, which might have impacted the results due to social desirability.

The study has statistical limitations. First, even though CFA testing of the ECBI’s internal structure was estimated with WLSMV, invariance models were tested with MLR estimation because some of the answer options were not selected by participants from some subgroups. Different results could have been expected if WLSMV was allowed. Lastly, the final internal structure was grounded on the data rather than driven by an expected theoretical framework. Hence, analyses were implemented in an exploratory mode, which is inconsistent with CFA and GRM. We tried to attenuate the bias by splitting the sample into two halves, of which one was tested by EFA and the other by CFA. Moreover, based on the results of the CFA, two items were removed. As this approach is questionable, the proposed internal structure still needs to be validated with other samples.

Lastly, not as much as a limitation but still noteworthy, the ECBI’s current form still needs to have further validity and reliability evidence identified. For instance, other measures of employability may be used to evidence the scale is assessing employability beliefs. Criterion validity could also be assessed by longitudinal studies investigating whether the ECBI’s scores may predict employability behavior (such as job search) or outcomes (such as being employed in the future). Lastly, time reliability still needs to be assessed by test-retest.

## Conclusions

This article introduces the psychometric properties of a brief version of the ECBI in a sample of unemployed persons. Validity evidence based on the internal structure and associations with related measures were identified yet discriminant validity was questionable. Reliability evidence based on internal consistency and measurement invariance was acknowledged. In addition to introducing the ECBI’s psychometric properties, this paper brings theoretical and methodological contributions. From the theoretical perspective, the findings describe a new theoretical framework on career and employability beliefs composed of three dimensions, i.e., growth, pessimism, and flexibility. From the methodological perspective, the article discusses the impact of statistical choices on the identification of psychological measures’ validity and reliability evidence.

## Figures and Tables

**Fig. 2:**
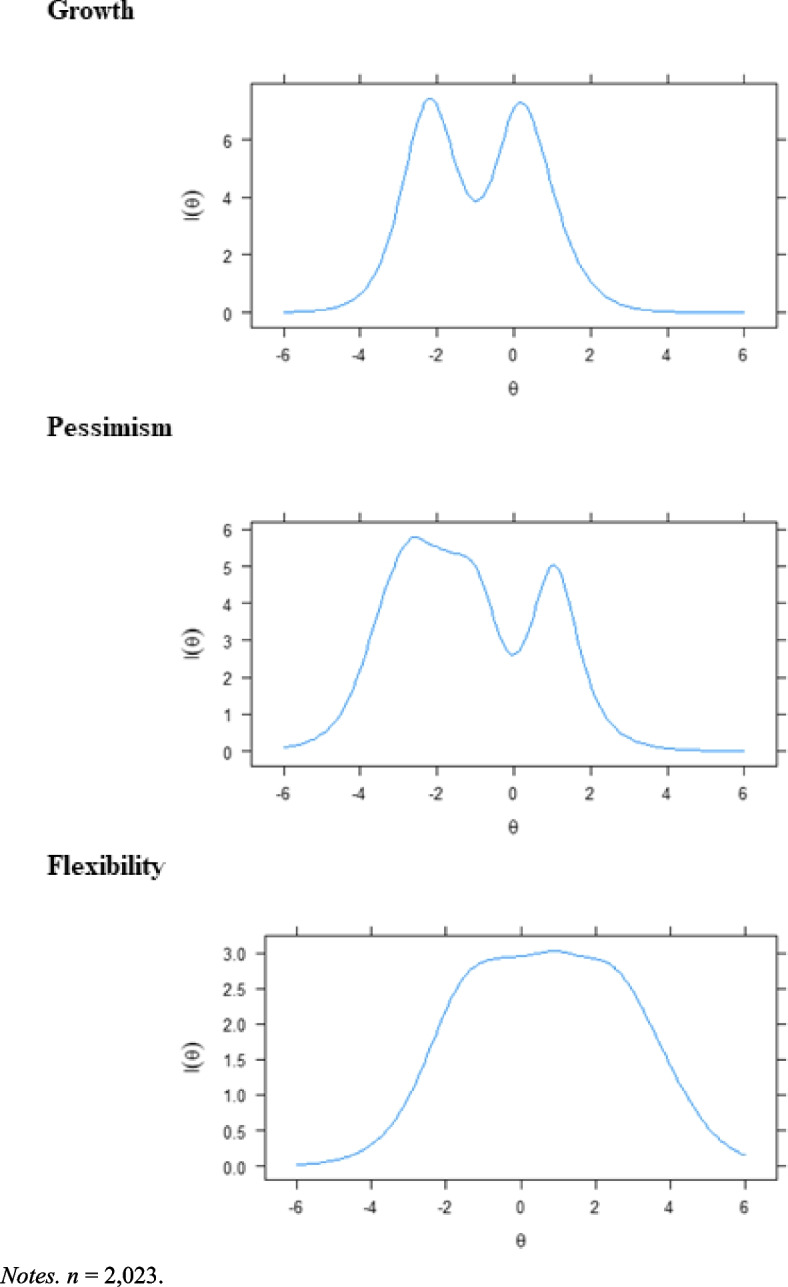
Test information curves. Note: *n* = 2023

**Fig. 3:**
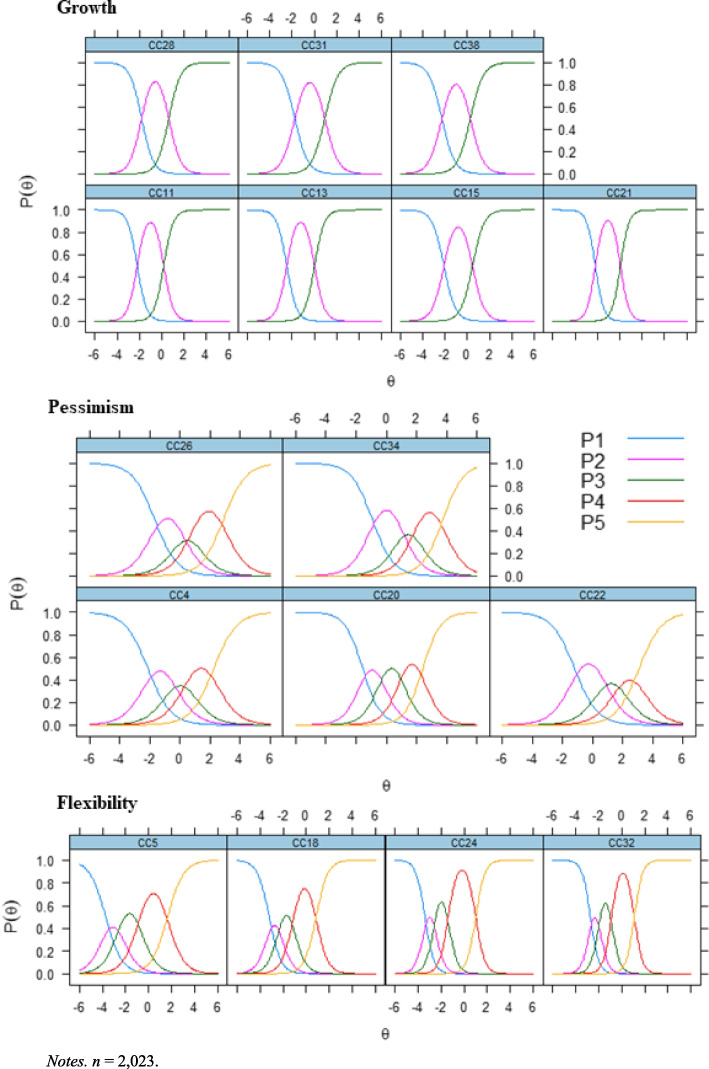
Item characteristic curves. Note: *n* = 2023

## Appendix 1

**Table 5:** GRM— monotonicity, item fit, and local dependence testing

Growth	Mon.	*S-χ*^*2*^ test	*Q*_*3*_ test						
	*H*	*RMSEA*	Item 11	Item 13	Item 15	Item 21	Item 28	Item 31	Item 38
Item 11	.52	.030	1.000						
Item 13	.53	.029	−.142	1.000					
Item 15	.49	.041	−.195^1^	−.086	1.000				
Item 21	.54	.028	−.181^1^	−.191^1^	−.195^1^	1.000			
Item 28	.51	.069	−.210^1^	−.146	−.054	−.167^1^	1.000		
Item 31	.52	.057	−.155	−.178^1^	−.141	−.122	−.079	1.000	
Item 38	.46	.057	−.060	−.196^1^	−.146	−.163	−.155	−.042	1.000
	Mon.	*S-χ*^*2*^ test	*Q*_*3*_ test						
Pessimism	*H*	*RMSEA*	Item 4	Item 20	Item 22	Item 26	Item 34		
Item 4	.35	.024	1.000						
Item 20	.39	.012	−.127	1.000					
Item 22	.35	.021	−.106	−.256^1,2^	1.000				
Item 26	.35	.024	−.207	−.268^1,2^	−.117	1.000			
Item 34	.36	.021	−.242^2^	−.203	−.187	−.106	1.000		
	Mon.	*S-χ*^*2*^ test	*Q*_*3*_ test						
Flexibility	*H*	*RMSEA*	Item 5	Item 18	Item 24	Item 32			
Item 5	.45	.021	1.000						
Item 18	.50	.028	−.165	1.000					
Item 24	.54	.030	−.182	−.228	1.000				
Item 32	.54	.027	−.175	−.292^2^	−.350^1,2^	1.000			

*n* = 2023, *Mon.* monotonicity, ^1^item pairs with residual correlation above |1/(*L*-1)|, ^2^item pairs with residual correlation above .224

## Appendix 2

**Table 6:** GRM—overall model fit, person fit, and reliability

	Overall model fit (*C*_*2*_ test)					Person fit	
	*C*_*2*_(*df*)	RMSEA [C.I. 90%]	*CFI*	*TLI*	*SRMR*	% (Zh < −.30)	*ρ*
Growth	63.918 (14)*	.042 [.032; .053]	.993	.990	.025	3.6%	.82
Pessimism	42.527 (5)*	.061 [.045; .078]	.983	.966	.039	1.9%	.74
Flexibility	2.714 (2)	.013 [.000; .048]	.999	.999	.048	2.5%	.78

Note: *n* = 2023, **p* < .01.

## Abbreviations

ECBI: Employability and Career Beliefs Inventory
PCA: Principal component analysis
EFA: Exploratory factor analyses
UniCo: Unidimensional congruence
ECV: Explained common variance
MIREAL: Mean of Item Residual Absolute Loadings
CFI: Comparative Fit Index
TLI: Tucker-Lewis Index
RMSEA: Root mean square error approximation
SRMR: Standardized root mean residual
GRM: Graded response model
ICC: Item characteristic curves
TIC: Test information curves
AVE: Average variance extracted

## Footnotes

1 Tested in a confirmatory factor analysis using WLSMV estimation method. The cutoff values are presented in the section Data Analysis. Even though RMSEA did not meet an acceptable value, Kenny et al. (2006) stated that RMSEA often malfunctions in models with reduced degree of freedom.

2 Four items of striving/achievement; two items of acceptance of challenges/risks; two items of optimism; five items of proactivity/initiative; one item of autonomy; and one item of flexibility/openness to change.
